# Influence of Hybrid Pedagogical Models on Learning Outcomes in Physical Education: A Systematic Literature Review

**DOI:** 10.3390/ijerph19159673

**Published:** 2022-08-05

**Authors:** Yafei Shen, Weide Shao

**Affiliations:** 1 College of Teacher Education, Zhejiang Normal University, Jinhua 321004, China; 2 College of Physical Education and Health Sciences, Zhejiang Normal University, Jinhua 321004, China

**Keywords:** hybridization, pedagogical models, learning outcomes, physical education

## Abstract

Hybrid implementation of pedagogical models (PMs) helps to overcome the limitations of a single pedagogical model (PM) when it comes to improving student learning outcomes in physical education (PE). Empirical research on hybridizations has grown substantially in recent years, so the purpose of this study was to conduct a systematic review on the effects and mechanisms of different hybridizations on students’ learning outcomes (i.e., motor, cognitive, affective, and social) in PE. Electronic databases, including ERIC, SCOPUS, EBSCO host, and Web of Science, were used to select intervention studies. After inclusion and exclusion criteria were applied, 17 high-quality studies, published in English peer-reviewed journals, were assessed. Results show that there were seven different hybrid models having impacts on students’ learning outcomes, which could be divided into four categories: (1) game performance and technical skills; (2) understanding of tactics and decision-making abilities; (3) motivation, autonomy, and confidence; (4) interpersonal skills, cooperative learning ability, and responsibility. Length of implementation and teachers’ familiarity were the main factors that limit the implementation on hybridizations. Future research should consider quasi-experiments with control groups of hybrids versus single models to figure out the advantages of the hybrid model over the single model; including more evidence from different schools, regions, and countries is necessary.

## 1. Introduction

PM hybridization refers to the combination of separate PMs or the respective components of each model [1,2,3]. Over the last decade, the phrase “models-based practice” (MbP) [4] has shaped the language of physical education [5,6,7]. Drawing upon the concepts of “curriculum model” [8] and “instructional models” [9], pedagogical models are acknowledged as a viable alternative to the conventional, teacher-centered approach (e.g., direct instruction) when implemented in complex school-based contexts [10]. Hastie and Casey [11] claim that a pedagogical model is “a blueprint which describes certain procedures for organizing content, task structures and the sequencing of learning activities” and each model has a specific design specification that prescribes the “non-negotiable” features that make it distinctive. Instead of primarily nouning/normalizing PMs, Casey et al. [7] agreed that they may also need to be described in a verbing or denormalizing process. In this way, models are regarded to be design specifications or frameworks [4], in which teachers are able to select the most effective method for delivering models in different local contexts, based on the teaching skills they deem most relevant. Further, it is acknowledged that pedagogical models are constantly modified and evolved with the goal of being produced, tested, polished, and further tested in alternative situations. Research on PE pedagogy includes a number of PMs that allow students to acquire breadth and depth of knowledge in PE in a variety of contexts [12]. In this regard, a range of pedagogical models were identified for one particular goal, for example, sport education (SE), aiming at developing competent, enthusiastic, and literate sports players [13,14], cooperative learning (CL), trying to increase social skills through group tasks [15], teaching personal and social responsibility (TPSR), enhancing students’ responsibility for their actions [16], health-based physical education (HBPE), encouraging healthy lifestyles [10], teaching games for understanding (TGfU) [17], with its variants step-game approach (SGA) and invasion games competence model (IGCM), belonging to game-centered models to provide students with opportunities to improve their skills in execution and decision-making. Accordingly, a myriad of research has identified pedagogical models as the most effective way to position students at the center of the teaching–learning process [9], allowing for the assessment of the impacts on students’ learning in PE. Extensive research has reflected the potential of PMs for achieving fundamental outcomes associated with PE: motor, cognitive, social, and affective skills [5,18,19,20,21].

Since each model is developed to focus on one certain curriculum goal, each model has its limitations when implemented in isolation [17,18], such as how students will experience failure when participating in competitive learning situations [6]. Therefore, focusing on building students’ social and emotional skills and capacity might help them better manage and deal with failure in PE [22]. In addition, it is widely acknowledged that no one model can accommodate all PE contexts. As a result, the above models were hybridized to fit the different educational frameworks. Both of these ideas derive from the same root, which may be described as the combination of separate PMs or the respective components of each of these ideas, and the term hybridization has been used to symbolize both of these ideas [3]. PMs are hybridized because they have similar characteristics and goals, which facilitates their hybridization. For example, previous studies have proved the feasibility of TPSR and SE hybridization by observing that the two models share the same learning theory [23], while SE and CL offer a situated learning context with meaningful activities [24]. Practical investigations have shown that TPSR and SE may be successfully combined as long as the implemented project intends to provide students with the opportunity to experience all five levels of responsibility in the framework season of SE [25,26,27]. In other words, hybridizations conducted among pedagogical models refer to extracting and merging the key features of two models or using one model as a foundation and adding other significant elements from the other. Previous studies indicated that the hybridizations could increase game performance and motor skills [26,28,29] and generate positive psychosocial consequences, such as enjoyment, the intention to be physically active and responsibility [24,26,30].

Despite this increase in hybrid model studies in recent years, specific reviews about pedagogical model hybridizations remain rare. In fact, just one systematic review conducted an integrated examination on the hybridizations of various PMs (conducted between 2000 and 2018) [3]. As the first review in this field, it primarily provided the basic information of PM hybridizations in PE, including hybrid types, study focus, participants and context, sport/content, length of the implementation, data sources and analysis, and outcomes. It acknowledged that hybridization is beneficial to students’ game-related skills and psychosocial variables. To extend the work of González-Víllora et al. [3], we investigated further the reasons and mechanisms for hybrid models to promote learning outcomes by carrying out an exhaustive review of the empirical studies of PM hybridizations (conducted between 2000 and 2022). In particular, seven empirical studies were published in the past four years, especially after the coronavirus disease 2019 (COVID-19) [31,32,33,34,35,36]. During COVID-19, students adopted online remote learning as the standard operating process for learning in PE, which leads to some negative effects, such as a reduction in physical activity; lack of social and emotional support for students; and decreased motivation of pupils to engage in sports [37,38,39]. Therefore, it is necessary to address these issues by considering effective pedagogical models of PE. Furthermore, to our knowledge, there is a lack of synthesis that summarizes the findings of empirical interventions that aim to demonstrate that the hybridization of PMs has the potential to increase learning outcomes among students. For all the considerations mentioned above, the purpose of this systematic review was to analyze the impacts and mechanism of hybrid model applications on students’ learning variables (i.e., motor, cognitive, affective, and social), which directly influence students’ participation and experiences in PE.

## 2. Method

### 2.1. Data Sources and Search Strategy

The systematic review followed the Preferred Reporting Items for Systematic Reviews and Meta-analyses guidelines (PRISMA) [40]. An exhaustive search was initiated through four electronic databases: ERIC, SCOPUS, EBSCO host, and Web of Science. The search covered all articles published until February 2022, using the Boolean operators (AND, OR) to combine the search terms “pedagogical model”, “curriculum model”, “instructional models”, “physical education”, and “hybrid*”.

**Figure 1:**
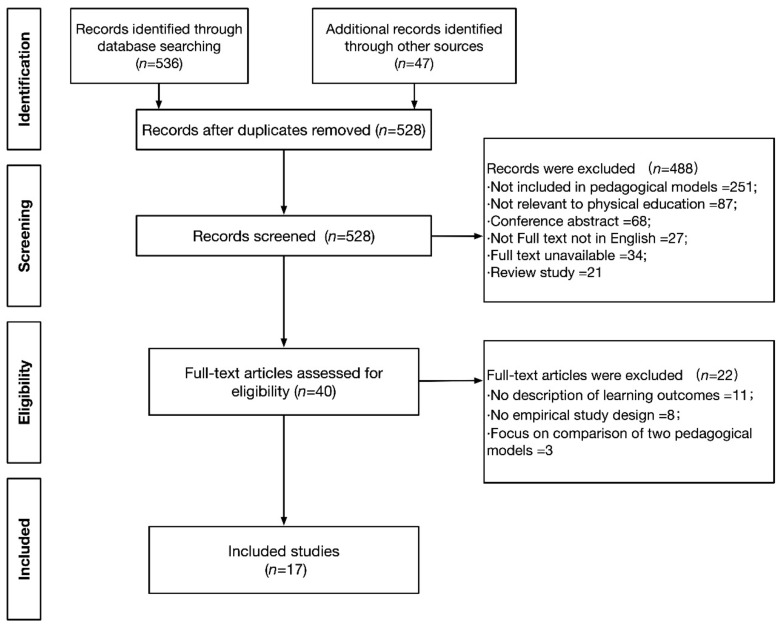
Flowchart of study selection process.

To reduce the possibility of selection bias, two experienced authors independently selected the studies. Both were familiar with PE pedagogical models. Initially, 536 publications were found using the search terms. Afterwards, the reference lists of the selected articles were screened for potentially relevant articles to include in the review (*n* = 47). After reading the titles and abstracts, the two researchers eliminated publications that did not meet the selection criteria, reviewed the full text individually based on the exclusion criteria, and summarized the final findings. Finally, 17 articles were included for systematic review and analysis (Figure 1).

### 2.2. Eligibility Criteria

According to the recommendation of Simonsohn et al. [41], the inclusion criteria for the literature were determined before electronic retrieval: (1) peer-reviewed journals published in international journals; (2) empirical studies performing the implementation of hybrid models in the PE context; (3) the main findings report at least one aspect of the learning outcomes of hybridizations on students; (4) articles published and written in English because this is the main language of the hybrid models; and (5) empirical studies using quantitative or qualitative or mixed research methods.

To ensure quality, books, book chapters, conference publications, master’s theses, and doctoral dissertations that had not been independently peer-reviewed were also eliminated. Meanwhile, studies published in non-peer-reviewed journals and/or not indexed in Journal Citation Reports (JCR) or Scientific Journal Rankings (SJR) were considered invalid. Following the study’s goal, comparison studies of different pedagogical models, the studies that did not specifically measure any aspect of learning outcomes, the studies conducted in the school-based PE content, and theoretical research were also excluded.

### 2.3. Data Extraction

Drawing on relevant published reviews in the field of PE pedagogical models [16,42], the following characteristics were summarized for each retained study: author(s)/country/year of publication, study focus, hybrid model implemented, length of the unit/content, participants, methodology, and main results (Table 1).

### 2.4. Quality Assessment and Level of Evidence

First, The PRISMA protocol was utilized to evaluate this systematic review’s quality. Second, the quality of the published manuscripts was assessed using a checklist adapted from the Strengthening the Reporting of Observational Studies in Epidemiology (STROBE) statement [43]. Nine assessment criteria were selected concerning the structure of a typical publication in this field of research: (1) description of PM hybridizations; (2) characteristics of the participants; (3) reasonable design of the study; (4) detailed data collection; (5) detailed data analysis; (6) data validity and reliability; (7) inclusion of models’ fidelity; (8) report of learning outcomes; (9) discussion of results. Accordingly, each item was scored 1 (yes) or 0 (no) points. The total quality score for each included study was determined by summing the individual scores. Studies were categorized as “high quality” if they scored 7 or more, “moderate quality” if they scored between 4 and 6 points, and “low quality” if they scored lower than 4. Manuscripts had to score at least 4 points to be selected. The results showed that 15 studies were identified as high quality and 2 studies were identified as moderate quality. There were no excluded low-quality studies (Table 2).

**Table 1:** Study characteristics.

Author/Year Country	Focus	Participants	Hybrid	Length of Unit/Content	Data Sources	Research Design & Analysis	Learning Outcomes
Hastie and Buchanan 2000 [44] United States	Examine the viability of SE-TPSR in action and develop a theory of Empowering Sport model	45 grade 6 boys; 11–13 years old	SE-TPSR	26 lessons; Xball	independent observations, daily debriefs, informal interviews	Qualitative: constant comparison technique	Social
Hastie and Curtner-Smith 2006 [1] Australia	Analyzing the impact of SE-TGFU implementation on teachers and students	29 grade 6 students (11 boys and 18 girls); 11–12 years old	SE-TGFU	22 lessons; batting/fielding games	Reflective log and notes critical incident reflective sheet; tactical quizzes; Game design forms; Team interviews	Mixed: analytic induction technique; enumerative analysis; typological analysis; constant comparison	Cognitive, affective
Mesquita et al., 2012 [45] Portugal	Analyze the impact of SE-IGCM on student decision-making, skill execution, and overall competition performance	26 grade 5 (17 girls and 9 boys); 10–12 years old	SE-IGCM	22 lessons; soccer	Game Performance Assessment Instrument (GPAI), Video observation	Quantitative: Mann-Whitney test, Wilcoxon test	Motor, cognitive
Stran et al., 2012 [46] United States	To examine pre-service teachers’ perceptions of SE-TGFU and analyze the facilitators and hindrances they experienced in implementing the model	22 pre-service teachers (14 male and 9 female, average age 23); 162 grade 5 students (10–11 years old)	SE-TGFU	20 lessons; Invasion games	focus group interviews, critical incident reflections, lesson plans, and observations.	Qualitative: thematic analysis method	Cognitive, affective
Farias et al., 2015 [47] Portugal	Analyzing the effects of SE-IGCM on students’ performance and game understanding in soccer games	24 grade 5 students (16 girls and 8 boys); mean age 10.3 years	SE-IGCM	17 lessons; soccer	The Game Performance Observation Instrument, Coding Association 6 Conference, The Game Understanding Test	Mixed studies: Mann-Whitney test, Wilcoxon test	Motor, cognitive
Araújo et al., 2016 [48] Portugal	Analyzing the impact of SE-SGA on student competition performance	17 grade 7 students (7 girls, 10 boys); average age 11.8 years	SE-SGA	25 class lessons; volleyball	Video Observation, Game Performance Assessment Tool (GPAI)	Quantitative: analysis of variance (ANOVA)	Motor, cognitive
Araújo et al., 2017 [49] Portugal	Examining the impact on student coaches’ pedagogical content knowledge (PCK)	21 students (11 males and 10 females); mean age 12.0	SE-SGA	20–25 lessons; volleyball	Video observation, field notes, interviews	Qualitative: thematic analysis method	cognitive
Fernandez-Rio and Menendez-Santurio 2017 [25] Spain	Assessing students’ and teachers’ perceptions of participating in taekwondo at SE-TPSR	71 grade 9 students; 15.4 ± 0.73 years old	SE-TPSR	16 lessons; Taekwondo	Open-ended questions, Photovoice, diaries of teachers and external observers, semi-structured interviews.	Qualitative: thematic analysis method	Social
Gil-Arias et al., 2017 [50] Spain	Measuring students’ motivation to participate in physical activity	55 grade 4 students (27 female, 28 male); mean age 15.45	SE-TGfU	16 lessons; volleyball	Scales: Autonomous motivation, Basic psychological needs, Enjoyment, Intention to be physically active	Quantitative: control group, MANOVA, Shapiro-Wilks test	Affective
Chiva-Bartoll, Salvador-García, and Ruiz-Montero 2018 [51] Spain	Examining the evolution of student motivational climate in physical education classes	96 grade 4 students; mean age 15	CL-TPSR	8 weeks; handball	Task Engagement Scale, Self-Engagement Scale	Quantitative: quasi-experimental designs, control groups, and	Affective
Araújo et al., 2019 [52] Portugal	Analysis of student competition performance in three SE-SGA seasons	18 grade 7 students (8 female and 10 male) 11–13 years old	SE-SGA	20–25 lessons; volleyball	Video observation, the Game Performance Assessment Instrument (GPAI),	Quantitative: hierarchical linear model	Motor
García-González et al., 2020 [31] Spain	Demonstrate whether SE-TGFU can be more effective for less motivated students	49 students (49% female, 51% male); mean age 15.50	SE-TGFU	10 lessons; volleyball	Basic Psychological Needs Support Questionnaire (BPNES), Basic Psychological Needs for Exercise Scale (BPNES), Novelty Needs Satisfaction Scale (NNSS), Perceived Variety of Exercise Questionnaire (PVE), Perceived Causality Questionnaire	Quantitative: anterior-posterior lateral measurements,	Affective
Gil-Arias, Diloy-Peña, et al., 2020 [33] Spain	Analyzing the effects of SE-TGFU on student motivational outcomes	53 grade 4 students (16 female, 37 male); mean age 15.50	SE-TGFU	10 lessons; volleyball	Questionnaires, focus groups	Mixed: one-way analysis of variance, analysis of variance, deductive content analysis	affective
Gil-Arias, Claver, et al., 2020 [32] Spain	Analysis of SE-TGFU on autonomy support, sensory Knowing the effects of motivating atmosphere, fun and perceptual ability	53 grade 4 students (16 female, 37 male); mean age 15.50	SE-TGFU	16 lessons; handball and basketball	Physical Education Class Learning and Performance Orientation Questionnaire, Autonomy Support Coaching Strategies Questionnaire, Enjoyment and Perceived Ability Scale	Quantitative: a counter-balanced crossover design	affective
Gil-Arias, Harvey, et al., 2020 [34] Spain	Investigating the effects of using SE-TGFU on perceived autonomy support, perceived need satisfaction, autonomy motivation, and adaptive outcomes	292 grade 6 students (140 female, 152 male); mean age 10.41	SE-TGFU	16 lessons; basketball	Autonomy Support Coaching Strategies Questionnaire, BPNs in Sport Scale, Perceived Causality Questionnaire, Relational Goals Questionnaire, Physical Activity Class Satisfaction Questionnaire	Quantitative: analysis of variance	Affective
Evangelio et al., 2021 [35] Spain	Explore students’ perceptions of the SE-CL-HBPE three-model mix	115 grade 5–6 students (46.09% girls); 10–13 years old	SE-CL-HBPE	13 lessons; an educative version of CrossFit (‘Edu-Crossfit’)	Interviews	Qualitative: thematic analysis method	Social
García-Castejón et al., 2021 [36] Spain	Effects on student health and psychosocial variables	99 students grade 1 and 2 of secondary school (51 girls and 48 boys); 12–14 years old	TPSR-TGfU	22 lessons; basketball, soccer, volleyball	Questionnaires, video recordings, semi-structured interviews	Mixed: a quasi-experimental pre-post study	Affective, social

**Table 2:** Quality score checklist.

Reference	Description of PM Hybridizations	Characteristics of the Participants	Reasonable Design of the Study	Detailed Data Collection	Detailed Data Analysis	Validity and Reliability	Inclusion of Models’ Fidelity	Report of Learning Outcomes	Discussion of Results	Total Score
Hastie and Buchanan 2000 [44]	1	1	1	1	1	1	0	1	1	8
Hastie and Curtner-Smith 2006 [1]	1	1	1	1	1	0	0	1	1	7
Mesquita et al., 2012 [45]	1	1	1	1	1	1	0	1	1	8
Stran et al., 2012 [46]	1	1	1	1	1	0	1	1	1	8
Farias et al., 2015 [47]	1	1	1	1	1	1	0	1	1	8
Araújo et al., 2016 [48]	1	1	1	1	1	1	1	1	1	9
Araújo et al., 2017 [49]	1	1	1	1	1	0	0	1	1	7
Fernandez-Rio and Menendez-Santurio 2017 [25]	1	1	1	1	1	0	0	1	1	7
Gil-Arias et al., 2017 [50]	1	1	1	1	1	1	0	1	1	8
Chiva-Bartoll, Salvador-García, and Ruiz-Montero 2018 [51]	1	1	1	1	1	1	0	1	0	7
Araújo et al., 2019 [52]	1	1	1	1	1	0	1	1	1	8
García-González et al., 2020 [31]	1	1	1	1	1	0	1	1	1	8
Gil-Arias, Diloy-Peña, et al., 2020 [33]	1	1	1	1	1	0	1	1	1	8
Gil-Arias, Claver, et al., 2020 [32]	1	1	1	1	1	0	1	1	1	8
Gil-Arias, Harvey, et al., 2020 [34]	1	1	1	1	1	0	1	1	1	8
Evangelio et al., 2021 [35]	1	1	1	1	1	1	0	1	1	8
García-Castejón et al., 2021 [36]	1	1	1	1	1	1	1	1	1	9

## 3. Results

### 3.1. Study Description

Study background. All of the 17 identified studies were undertaken in a Western country, with the majority of them implemented in Spain (9), Portugal (5), the United States (2), and Australia (1). The first article in this area was published in 2000 [44], with an increasing trend over the years.

Participants and content implemented. All studies investigated the effects of a hybrid pedagogical model on elementary and secondary school pupils in PE classes. The total sample of students in these 17 articles was 1127; most students were between the ages of 10 and 15. Regarding the content, the physical education program is based on ball sports (including football, basketball, volleyball, and handball), taekwondo, and frisbee.

The types of hybrid models. Sixteen papers conducted a hybridization of two PMs, whereas one paper used a hybridization of three PMs (SE-CL-HBPE) [37]. The greatest hybridization of the two PMs, with 7 articles, is SE-TGFU hybridization. The rest are SE-SGA (3), SE-IGCM (2), SE-TPSR (2), CL-TGfU (1), and TPSR-TGfU (1). In summary, there are seven types of hybrid models. Most hybrid types are “SE+ one models”, where the unit’s organizational structure is based on the season of SE. Besides, SE is aimed at helping students become enthusiastic, competent, and literate sports players by providing a meaningful sports experience [53]. The learning tasks and the content to be taught during the season mainly come from game-centered models (TGFU, IGCM, and SGA) and TPSR. As a derivation of TGFU, IGCM and SGA prioritize developing students’ technical and tactical abilities in offensive and defensive sports (IGCM) and net sports (SGA) to assure game success [45,48]. TPSR promotes personal and social responsibility among students, increasing their accountability for their activities [16]. CL seeks to strengthen students’ social skills via group work [54], while HBPE encourages team members to appreciate their own physically healthy lives by encouraging healthy lifestyles [10].

Length of unit: The implementation time ranged between 10 and 26 lessons, and 13 hybridizations with SE were conducted in a single hybrid season, with the exception of one that proceeded across three successive seasons [52].

Data collection and study design. Different research designs are used for intervention. Qualitative research mostly collects data via interviews and observations, while quantitative research primarily collects data using scales and questionnaires. There is a total of eight quantitative studies, six qualitative studies, and three mixed research. All the studies are of high quality.

### 3.2. The Impact of Hybridizations on Students’ Learning Outcomes

#### 3.2.1. The Impact of Hybridizations on Students’ Motor Learning

This section is intended to demonstrate how learning in motor domains has been observed. It is stated that motor learning has been primarily positioned as physical growth (physical characteristics and technical skills). Specifically, the literature revealed that two PM hybridizations, SE-IGCM and SE-SGA, affected students’ skills and game performance. Mesquita et al. [45] investigated the effect of SE-IGCM on students’ skill levels and game performance. They discovered that the framework of learning assignments IGCM gave students opportunities to enhance their skill execution. Farias et al. [47] expanded on this by studying the growth of SE-IGCM on students’ game performance and game comprehension using pre-and post-tests and constructing a relationship between the two, indicating that a hybrid of SE-IGCM was able to enhance students’ game performance and knowledge. However, by analyzing the interview results of teachers, García-Castejón et al. [36] discovered that the amount of time spent on physical activity was significantly decreased as a consequence of teachers having to devote a great deal of time to explanations.

Some studies examine the link between gender and skill level and reach contradictory conclusions. Three-year longitudinal research (covering three seasons) assessed the competition performance of SE-SGA middle-school pupils and discovered that both male and female students’ skill levels had increased [52]. Nonetheless, some studies [45,47] also showed that the hybridization of PMs had enhanced the overall ability level of girls. In the research on the application of SE-SGA conducted by Araújo et al. [48], it was discovered that girls had more benefits in acquiring specific skills and tactics. In addition, the research indicates that hybridizations boost the ability level of boys more than girls [49]. The impact of hybridizations on the sports skills and competitive performance of both sexes must, thus, be investigated further.

In addition, it is considered that low-skilled students are the primary benefactors of hybridizations [49], confirming the findings of earlier research on SE and SGA [48]. This may be caused by two factors: first, higher-skill-level students may have been hampered by a ceiling effect; second, higher-skill-level students may have required more challenging tasks to guarantee that all students worked within the “zone of proximal development” [52].

#### 3.2.2. The Impact of Hybridizations on Students’ Cognitive Learning

This part aims to explain how cognitive learning is observed. Cognitive learning is mainly about tactics and decision-making abilities [55], which are significant components in games teaching. In particular, the literature demonstrated that four PM hybridizations (i.e., SE-TPSR, SE-TGFU, SE-IGCM, and SE-SGA) reported the results of pedagogical content knowledge (PCK) and tactical decision-making. Both early studies reported positive effects on students’ tactical understanding and tactical decision-making ability [1,44]; Mesquita et al. [45] combined the variation IGCM of TGFU with SE to improve students’ tactical decision-making skills. Araújo et al. [49] studied the development of PCK throughout the three SE-SGA seasons. After particular intervention measures, the results indicated that student coaches increased their abilities to arrange and introduce tasks to teammates, recognize skill faults, offer feedback, and alter assignments for various team members.

#### 3.2.3. The Impact of Hybridizations on Students’ Affective Learning

In the original studies included in this paper, affective learning typically included psychological factors, such as self-confidence, self-esteem, motivation, and a sense of self-worth [56,57]. There are five hybrid programs, SE-TGfU, SE-TPSR, CL-TPSR, TPSR-TGfU, and SE-CL-HBPE, reporting positive results. From the perspective of physical education instructors, two qualitative investigations found that students were more involved with the SE-TGfU and their learning in the affective domain increased [46,58]. By examining three hybrid models (SE, CL, and HBPE), Evangelio et al. [35] discovered that student autonomy was improved and that group work and role playing successfully boosted students’ confidence and sense of achievement.

Motivation is a hot topic in the field of affective learning. The research results agree that the degree of motivation is the crucial factor affecting students’ participation in physical activities. Gil-Arias et al. [50] experimentally discovered that participation in the SE-TGfU led to significant improvements in student autonomy, competence, and enjoyment. In five studies framed by self-determination theory, the CL-TPSR promoted students’ team participation, understanding of the game, teamwork, enjoyment, and involvement [51]. TPSR-TGfU showed significant increases in students’ willingness to engage in physical activity, autonomous motivation, self-determination, psychologically mediated regulation, personal and social responsibility, enjoyment, and significant decreases in negative emotions. Three studies were conducted on the effects of the SE-TGFU on student motivation, with two utilizing volleyball instruction [31,33] and one utilizing basketball instruction [34]. Garca-González et al. [31] concluded that the SE-TGFU improved student motivation, particularly for those students who displayed low to moderate levels of motivation at the beginning of the intervention; Gil-Arias, Diloy-Peña, et al. [33] and Gil-Arias, Harvey, et al. [34] agreed that the hybrid model had a greater impact on girls’ psychological needs satisfaction, novelty and diversity acquisition, and intrinsic motivation.

#### 3.2.4. The Impact of Hybridizations on Students’ Social Learning

Typically, social learning encompasses (a) interpersonal skills; (b) interpersonal relationships and the ability to listen to team members; and (c) beliefs, idea sharing, and co-constructing new understandings [54,57,59]. According to studies, social learning appears to benefit from two hybrid programs, SE-TPSR and SE-CL-HBPE. Three studies on SE-TPSR, exploring students’ social interactions, showed significant increases in students’ social responsibility and relationships after the intervention. Specifically, it was stated that the SE-TPSR was adequate for students to collaborate, develop self-esteem, and maintain social interactions [44]. This view is supported by Fernandez-Rio et al. [25], who demonstrated that SE-TPSR enables students to assume greater autonomy, gives them greater responsibility for their actions, and teaches them to pay attention to their rights, feelings, and the needs of others. Through three hybrid curricular models (SE, CL, HBPE), Evangelio et al. [35] found an increase in students’ cooperation and personal responsibility.

## 4. Discussion

Previous research demonstrated that single PM has limitations because it cannot meet all curriculum goals [3]. The aforementioned findings suggest that the hybrid model has an effect on students’ motor, cognition, affective, and social learning. In this section, we managed to figure out the mechanisms that can support the different hybrid implementations of PMs to achieve four learning outcomes.

### 4.1. The Mechanism of Hybridizations on Students’ Learning Outcomes

#### 4.1.1. The Mechanism of Hybridizations on Students’ Motor Learning

According to the results, the two hybridizations, SE-IGCM and SE-SGA, increased the motor skills and gaming performance of pupils. The reason is that SE, IGCM, and SGA are game centered and demand a certain level of motor skills from students. Therefore, interventions through hybridizations can help in the improvement in students’ motor skills and game performance. Previous studies reported the positive effects of SE, IGCM, and SGA in isolation on students’ motor learning [45,60,61]. SE aims to cultivate capable, educated, enthusiastic athletes by adopting more democratic and inclusive teaching methods. Its primary focus is on developing the organizational structure and authenticity of the learning environment [62,63], but it lacks specialized teaching strategies for developing students’ capacity to compete in tactics [63,64]. As a derivative of TGFU, IGCM and SGA emphasize providing an appropriate framework for the development of students’ technical and tactical skills in offensive and defensive sports (IGCM) and net sports (SGA) in order to ensure the success of the games [45,48]. Therefore, when SE is combined with IGCM and SGA, the model retains the main characteristics of SE, such as stable team, formal competition, and role play, while adding learning tasks from IGCM and SGA and sports skills to be taught during the season. Therefore, their combination allows students to increase their sports skill acquisition and performance in competition.

#### 4.1.2. The Mechanism of Hybridizations on Students’ Cognitive Learning

The above findings indicate that SE-TPSR, SE-TGFU, SE-IGCM, and SE-SGA can enhance students’ tactical understanding and decision-making skills. The main reason is that these four PMs are all game-based models. Previous studies in soccer [65], volleyball [66,67], badminton [68], basketball [69], and floorball [70] confirmed that knowledge of the game, comprehension of tactics, and capacity to make decisions were significantly higher in game-centered PMs compared with technical approaches. SE helps students comprehend real competition by placing them in different roles [44]. Therefore, SE mainly focuses on the organizational structure and authenticity of the learning environment, does not focus on the understanding of tactics and the implementation of skills, and lacks specific teaching strategies to develop students’ ability to compete in tactics [71]. In contrast, TGFU focuses on students’ capacity to comprehend the game: their tactical decision-making and skill execution [50]. Students might create tactical awareness and decision-making skills of the game by decreasing the technical requirements via appropriate modifications [72]. As the derivatives of TGFU, IGCM and SGA emphasize providing an appropriate framework for developing students’ technical and tactical abilities in game, such as invasion games and net/wall games, to achieve the success of the game [45,48].

#### 4.1.3. The Mechanism of Hybridizations on Students’ Affective Learning

In the aspect of affective learning, there are more hybrid forms of curriculum models, including five approaches: SE-TGfU, SE-TPSR, CL-TPSR, TPSR-TGfU, and SE-CL-HBPE. All five hybrid approaches promote the development of affective aspects, such as students’ self-confidence, self-esteem, motivation, and sense of self-worth. The primary reason for this is that all five models emphasize the importance of teamwork in motivating and inspiring students through collaborative team learning. Previous research demonstrated that SE, CL, and TGfU are founded on constructivist learning theories and emphasize contextual learning, providing students with enough opportunity to learn in an autonomous and supportive environment [16,48,73]. Students feel a sense of achievement through positive connections with their surroundings, which boosts their self-awareness and self-efficacy, increasing their motivation, autonomy, and physical activity engagement [50]. TPSR can improve pupils’ basic psychological requirements by emphasizing personal and societal responsibility. Once students’ fundamental psychological needs are satisfied, they can improve their motivation and chances to engage in physical activity outside physical education classes [74]. Thus, combining these five modalities can foster greater self-determined motivation and provide positive affective and emotional outcomes, such as the intention to enjoy and actively engage in physical activity, thereby enhancing students’ willingness to exercise.

#### 4.1.4. The Mechanism of Hybridizations on Students’ Social Learning

The findings above indicate that the two models, SE-TPSR and SE-CL-HBPE, can develop students’ social interaction abilities. It is mainly because both models emphasize cooperative learning and student involvement in teaching and learning. Firstly, responsibility is one of the key elements of TPSR, helping students to promote personal and social responsibility, giving them more responsibility for their actions, and teaching them to be sensitive to the rights, feelings, and needs of others. In addition, in SE, students are responsible for playing multiple roles, giving them opportunities to practice personal and social responsibility. Consequently, the similarities between the two models can positively impact students’ personal and societal responsibility [25,26,44]. When SE is hybridized with TPSR, individuals attempting to develop personal responsibility in SE are put in a social environment, in which their actions have consequences for others. Meanwhile, the TPSR teaching techniques, such as building awareness and reflection and adopting goal levels, were able to fit well within the framework of an SE season and helped to improve the players’ responsibility in SE. This result also validates the high compatibility in the single implementation of SE and TPSR found in earlier research [23,75]. Second, SE puts particular emphasis on long-term, stable teams [76]; CL attempts to develop students’ social skills through group work [54]; and HBPE encourages team members to value their own physically healthy lifestyles by promoting healthy lifestyles [10]. Small-group instruction and practice require students to depend on one another to accomplish the learning task. Given the presence of “team and group” in each hybrid model, students are better equipped to learn how to operate in teams and improve their social relationships.

### 4.2. The Limitations of Implementation on Hybridizations

First, the length of implementation is quite limited. It is mainly manifested in two aspects: (1) It is challenging to complete the requirements of more than two modes in a limited time. For instance, Hastie and Curtner Smith [1] devoted the majority of their efforts to teaching primary class agreements, team roles, and duties in an SE model and how to perform independent team activities. In the limited duration of 30 min, the teaching content of TGFU is often cut off. (2) The previous research on the teaching intervention time of PM hybridizations is usually short, which does not allow for sufficient time to regulate all variables that may interfere with the teaching process, resulting in an imprecise evaluation of the impact on students [51,52]. In conclusion, the combination of the two models places significant pressure on the completion of instructional activities.

The second is increased teaching requirements for PE teachers. Using hybridizations is a challenging teaching task that requires a comprehensive understanding of the theories, methodologies, and procedures of various modes, as well as teaching experience with multiple modes [46,77]. Although all teachers have had the experience of teaching a single-curriculum model in this study, researchers point out that the limited experience and knowledge of pre-service teachers on hybridizations has led to significant obstacles in implementation [46]. Moreover, they are unfamiliar with the student-centered method, the complex and changeable teaching environment [78], as well as the difficulties encountered in developing hybridizations. Teachers are rarely able to implement hybridizations in depth or for a long time, which is particularly common among novice teachers [26,33,46,49]. This finding emphasizes the importance of early training for PE teachers using hybridizations.

We suggest that PETE should consider the following aspects in implementing hybridizations. Due to their conflicting aims, implementing two models simultaneously in PE classrooms may result in tensions. For instance, when it comes to the usage of games under SE-TPSR, SE strives to encourage good sportsmanship, but TPSR wants to assist youth to become better individuals [25,44]. Rather than striving to achieve the objectives of both models, a choice must be taken as to which model should take precedence when implemented. In addition, the content and learning task should be adjusted to meet different-skill-level students.

## 5. Conclusions

This systematic review examined the effect of the hybridization of PMs on the learning outcomes of students. As PMs have similar characteristics or objectives, their hybridization is made possible. In other words, hybridizations of pedagogical models relate to the extraction and integration of essential characteristics from two models, or the use of one model as a base and the addition of other crucial parts from the other. The findings indicate that hybridization can facilitate children’s and adolescents’ learning in the motor, cognitive, affective, and social domains. The hybridizations might improve game performance and motor abilities and provide good psychological outcomes, such as enjoyment, the intention to be physically active, and responsibility. This article was also able to investigate the mechanisms that enable the different hybrid implementations of PMs to achieve four learning outcomes. Hybrid PMs possess the characteristics of multiple models and overcome the constraints of the individual model. There are currently seven hybridizations available internationally and their intervention studies require further exploration. 

Despite the aforementioned advantages, there are several limits and future research directions to consider. First, this paper explored the exteroception-based approaches of mechanisms, such as game understanding, skill execution, competition performance, pedagogical content knowledge, and personal and social responsibility. More experimental studies are encouraged to be conducted on the correlation between learning outcomes and interoception, which refers to the sense of the internal state of the body senses. Second, future research should employ diverse and innovative interventions to address instructional length and teacher capacity to develop PM hybridizations more effectively and obtain more experimental evidence. Specifically, it is required to replicate the present research and examine the influence on learning outcomes over a more longitudinal time frame by applying continuous units. In terms of methodology, future research could adopt more objective sampling methods, such as random and stratified sampling; increase sample sizes to include more evidence from various schools, regions, and countries; and consider quasi-experiments with control groups of hybrids versus single models to establish the advantages of the hybrid model over the single model. This would allow for a more robust evaluation of the hybrid models.
